# Five-Year Results of Aortic Remodeling for Acute, Subacute, and Chronic Type B Aortic Dissection Following Endovascular Repair

**DOI:** 10.3389/fcvm.2022.847368

**Published:** 2022-05-17

**Authors:** Guangmin Yang, Hongwei Ge, Guangyan Wu, Yepeng Zhang, Leiyang Zhang, Ming Zhang, Xiaoqiang Li, Min Zhou

**Affiliations:** ^1^Department of Vascular Surgery, Drum Tower Hospital, Affiliated to School of Medicine, Nanjing University, Nanjing, China; ^2^Department of Thoracic and Cardiovascular Surgery, Nanjing First Hospital, Nanjing Medical University, Nanjing, China; ^3^Department of Vascular Surgery, The Third Affiliated Hospital of Soochow University, Changzhou, China

**Keywords:** multicenter-center retrospective cohort study type B aortic dissection, thoracic endovascular aortic repair, aortic remodeling, endoleak, type B aortic dissection

## Abstract

**Background:**

This study was performed to compare aortic remodeling and clinical outcomes in patients with acute, subacute, and chronic type B aortic dissection (TBAD) after thoracic endovascular aortic repair (TEVAR).

**Methods:**

We retrospectively examined 323 consecutive patients with acute (*n* = 129), subacute (*n* = 161), and chronic (*n* = 33) TBAD who underwent TEVAR from June 2013 to December 2016 in in multicenter institution. Patient demographics, clinical data, and imaging characteristics were recorded and compared among the three groups.

**Results:**

The three groups had comparable baseline characteristics. Perioperative mortality rates were similar among the acute (2.3%), subacute (0.0%), and chronic (0.0%) groups (*P* = 0.34). Perioperative morbidity rates, including the rates of visceral and lower limb malperfusion and cerebral infraction, were also similar. The rate of perioperative endoleak was significantly higher in the chronic group (18.1%) than in the acute (3.9%) and subacute (3.7%) groups (*P* = 0.02). The mean follow-up duration was 78 ± 22 months (range, 36–101 months). The mortality rates were comparable among the three groups. The rates of reintervention and lower limb malperfusion were higher in the chronic group than in the acute and subacute groups. FL diameter reduction were more robust in the acute and subacute groups than in the chronic group.

**Conclusion:**

Patients with acute, subacute, and chronic TBAD had different outcomes in this study. Patients with acute and subacute TBAD had fewer complications than those with chronic TBAD. Aortic remodeling after TEVAR was more favorable in patients with acute and subacute TBAD than in patients with chronic TBAD. TEVAR promotes more positive remodeling at the stent graft level than at the distal level of the aorta.

## Highlights

- Type of research: Multicenter-center retrospective cohort study.
- Key Findings: The 323 patients with type B aortic dissection who underwent thoracic endovascular aortic repair had excellent outcomes. Patients with acute and subacute TBAD had fewer complications than those with chronic TBAD. Aortic remodeling after TEVAR was more favorable in patients with acute and subacute TBAD than in patients with chronic TBAD.
- Take Home Message: TEVAR promotes more positive remodeling at the stent graft level than at the distal level of the aorta.

## Introduction

Aortic dissection is the most common catastrophic disorder of the aorta and is associated with high mortality. Complicated type B aortic dissection (TBAD) is generally treated with thoracic endovascular aortic repair (TEVAR). Uncomplicated TBAD is treated medically until aorta-related adverse events occur. However, approximately one-third of patients with medically treated TBAD will require endovascular or open aortic repair within 5 years (1, 2). TEVAR closes the primary entry tear of the dissection, restoring blood flow to the true lumen (TL) and depressurizing the false lumen (FL). Its long-term objective is positive aortic remodeling, which involves expansion of the TL, thrombosis of the FL, and stabilization or regression of the overall aortic size. Previous studies of TEVAR for TBAD have mainly compared and analyzed patients in the acute and chronic phases. Aortic remodeling after TEVAR is more prominent with acute than chronic dissection (3, 4). However, acute dissection is associated with higher complication and mortality rates (5, 6). Nonetheless, the overall treatment outcomes are similar (7, 8).

The optimal timing of TEVAR to maximize aortic remodeling while minimizing the risk of complications remains unknown. Aortic remodeling and outcomes after TEVAR in patients with subacute dissection have not been well studied. Furthermore, differences in postoperative aortic remodeling among the three phases have not been examined. Therefore, this study was performed to investigate morphological changes in the aorta and postoperative outcomes in patients with acute, subacute, and chronic TBAD who undergo TEVAR.

## Materials and Methods

### Study Design and Patient Selection

This retrospective non-randomized multicenter study included 323 consecutive patients who underwent TEVAR for TBAD from June 2013 to December 2016 at Nanjing Drum Tower Hospital (Nanjing, China), The People’s Hospital of Changzhou (Changzhou, China), and Nanjing First Hospital (Nanjing, China). The patients were categorized into acute, subacute, and chronic groups according to the timing of TEVAR. Acute was defined as treatment within 14 days of symptom onset, subacute was defined as treatment from 2 weeks to 3 months of symptom onset, and chronic was defined as treatment after 3 months of symptom onset (9). Patients with connective tissue disease, aortitis, residual type A aortic dissection, traumatic aortic dissection, and atypical aortic dissection (such as a penetrating aortic ulcer) were excluded.

All patients received medical therapy. The indications for TEVAR were complicated dissection, a >10-mm increase in the aortic diameter per year, acute dissection with a maximal aortic diameter of >40 mm and a patent TL and FL, and subacute or chronic dissection with a maximal aortic diameter of >55 mm. Complicated dissection was defined as the presence of aortic rupture, hemothorax, malperfusion syndrome (visceral, renal, or acute lower limb), refractory hypertension, or persistent pain for >3 days.

All patients underwent computed tomography angiography (CTA) of the chest, abdomen, and pelvis at presentation to evaluate the TL, FL, and size and precise location of the entry tear. Postprocedural CTA was evaluated for endoleak, retrograde type A dissection, stent-induced new entry, and remodeling of the FL. The TL and FL diameters were measured in three-dimensional multiplanar reconstruction mode in the plane perpendicular to the centerline of the aorta at the following four levels of the aorta: proximal thoracic aorta 2 cm below the left subclavian artery (LSA) ostium, tracheal protuberance, diaphragm, and highest renal artery level.

### Thoracic Endovascular Aortic Repair

Thoracic endovascular aortic repair was performed with the patients under general anesthesia, and the goal was to provide complete coverage of the primary entry tear to induce stasis of blood flow and thrombosis in the FL. The stent graft was selected according to the diameter of the proximal landing zone. Grafts were sized up to a maximum of 10% larger than the diameter of the native aorta in the proximal landing zone. The extent of stent graft coverage was in the thoracic aorta above the level of the diaphragm in all patients. The endografts used in this study included the Ankura (LifeTech, Shenzhen, China) in 119 patients, Valiant (Medtronic, Inc., Minneapolis, MN, United States) in 108 patients, Zenith TX2 (Cook Medical, Bloomington, IN, United States) in 28 patients, TAG (W. L. Gore, Flagstaff, AZ, United States) in 43 patients, and Hercules (MicroPort Endovascular MedTech, Shanghai, China) in 69 patients. Adjustive treatments and techniques, including the chimney technique, single-branched stent technique, *in situ* fenestration technique, and carotid–subclavian bypass, were performed if the dissection involved the aortic arch or an effective proximal landing zone was not present.

### Outcomes and Definitions

**FIGURE 1:**
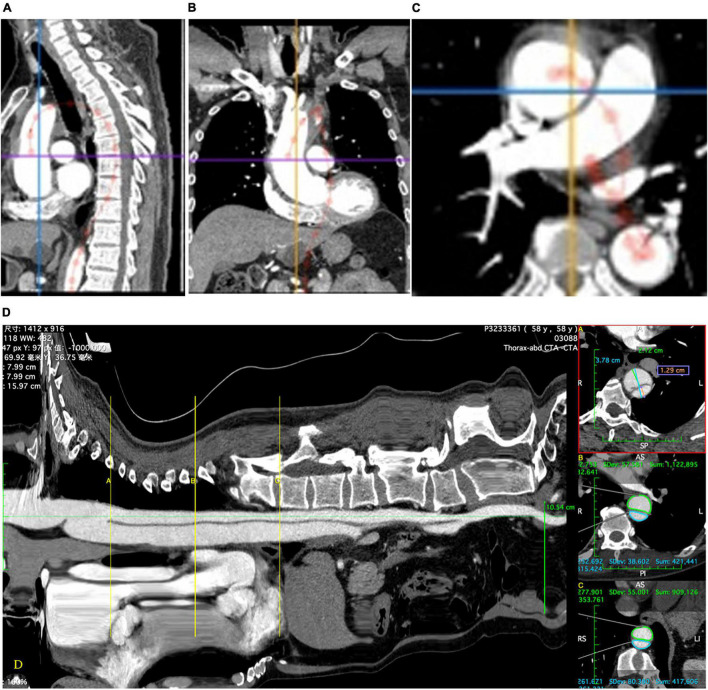
The central luminal line is installed which allows a “stretch view” to be produced **(D)**. This allows accurate determination of anatomic landmarks and precise location of measurements. Diameters **(A)** and areas **(B,C)** are measured using multiplanar reconstructions of the computed tomographic images in the plane perpendicular to the central luminal line using the appropriate measurement tools **(D)**.

The primary outcome was the change in the aortic diameter on CTA after TEVAR (postoperative diameter – preoperative diameter). Method of true and FL diameter measurement: the distance between the TL and FL perpendicular to the inner diaphragm (Figure 1A). Method of area measurement: the area of the TL and FL were measured by drawing around the corresponding lumens (Figures 1B–D). Positive aortic remodeling was defined as a 5-mm decrease in the maximum diameter of the thoracic aorta; an increase of >5 mm was defined as “progression,” and a change of ≤5 mm was defined as “no remodeling.” Technical success was defined as implantation of the stent graft with coverage of the primary entry tear, reperfusion of the TL, and no proximal type Ia endoleak. Major adverse events were defined as death, aortic rupture, visceral malperfusion, cerebral infarction, lower limb malperfusion, multiple organ dysfunction syndrome, spinal cord injury (SCI), and unintended reintervention. These adverse events are reported as perioperative (<30 days) and late.

### Follow-Up

Follow-up consisted of CTA at 1, 6, and 12 months, and yearly thereafter.

### Statistical Analysis

Continuous variables with a normal distribution are presented as mean with standard deviation and were compared using Student’s *t*-test. Continuous variables with a non-normal distribution are presented as median with interquartile range and were compared with the non-parametric test. Categorical variables are presented as number with frequency or percentage and were compared using the chi-square test or Fisher’s exact test. Statistical analyses were performed using SPSS software version 22.0 (IBM Corp., Armonk, NY, United States). A *P* value of <0.05 was considered statistically significant.

## Results

### Patient Characteristics

The patients’ characteristics are summarized in Table 1. Their mean age was 59.5 ± 13.7 years, and 293 (90.1%) were men. The number of patients classified in the acute, subacute, and chronic groups was 129, 161, and 33, respectively. The mean age, sex distribution, and prevalence of hypertension, coronary artery disease, peripheral vascular disease, renal failure, smoking history, and history of stroke were not significantly different among the groups. However, the prevalence rates of chronic obstructive pulmonary disease and diabetes were significantly higher in the chronic group (*P* = 0.02). Two patients (6.1%) in the chronic group had a history of previous TEVAR.

**TABLE 1:** Baseline clinical data of acute, subacute, and chronic TBAD patients.

	Acute (*n* = 129), *N* (%) or mean ± SD	Subacute (*n* = 161), *N* (%) or mean ± SD	Chronic (*n* = 33), *N* (%) or mean ± SD	*P*-value
**Demographic**				
Age, year	58.1 ± 7.5	60.2 ± 9.5	60.9 ± 9.7	0.15
Male	117 (90.1)	147 (91.3)	29 (84.8)	0.20
**Risk factors**				
Hypertension	102 (79.1)	133 (82.6)	27 (81.8)	0.30
Diabetes	10 (7.8)	11 (6.8)	4 (12.1)*	**0.03**
Hyperlipidemia	10 (7.8)	13 (8.1)	3 (9.1)	0.12
Coronary artery disease	6 (4.7)	5 (3.1)	1 (3.0)	0.21
Peripheral vascular diseases	5 (3.9)	4 (3.1)	1 (3.0)	0.31
COPD	10 (7.8)	14 (8.7)	4 (12.1)*	**0.02**
Stroke	6 (4.7)	7 (4.3)	2 (6.1)	0.31
Renal failure	3 (2.3)	3 (2.5)	1 (3.0)	0.42
Smoking	14 (10.9)	15 (9.3)	4 (12.1)	0.21
Refractory hypertension	25 (19.4)	33 (20.4)	1 (3.0)	0.42
Intractable pain	118 (91.5)	157 (97.5)	3 (9.1)*	**0.02**
Visceral malperfusion	13 (10.1)	1 (0.6)	0	0.13
Lower limb ischemia	9 (7.0)	2 (1.2)	1 (3.0)*	**0.01**
Previous TEVAR history	4 (3.1)	3 (1.9)	2 (6.1)*	**0.04**
Subtype class				
III a	81 (62.8)	98 (60.9)	12 (36.4)	0.39
III b	48 (37.2)	63 (39.1)	21 (63.6)	0.52

*COPD, chronic obstructive pulmonary disease; TEVAR, thoracic endovascular aortic repair.*

*Statistically significant difference between this group and either of the other groups. Boldface value, show P < 0.05.

### Procedural Details and Perioperative Outcomes

The overall technical success rate was 95.4% (308/323). Landing zone 3 was used in 288 (89.1%) patients. Landing zone 1 or 2 with an adjustive technique was used in the remaining 35 (10.9%). Among these patients, the LSA or left common carotid artery chimney technique was used in 22, LSA single-branch stent implantation in 9, and LSA–left common carotid artery bypass in 4. In total, 450 endografts were implanted. The mean proximal stent diameter was 33.7 ± 3.0 mm, and the mean oversize was 9.2 ± 1.7%. The mean stent graft length was 192 ± 25 mm.

Three perioperative deaths occurred in the acute group (2.3%) because of ongoing hemorrhage from a ruptured dissection, a ruptured dissection, and renal failure, respectively. No perioperative deaths occurred in the subacute and chronic groups. The incidence of perioperative major adverse events did not significantly differ among the acute (17.1%), subacute (16.1%), and chronic (18.2%) groups. Myocardial infarction, SCI, and retrograde type a dissection did not occur. Visceral malperfusion occurred in seven patients: three (2.3%) in the acute group, four (2.5%) in the subacute group, and none in the chronic group. The difference among the groups was not significant. Lower limb malperfusion also occurred in seven patients: three (2.3%) in the acute group, three (1.9%) in the subacute group, and one (3.0%) in the chronic group. The difference among the groups was not significant. Cerebral infarction occurred in 14 patients: 6 (4.7%) in the acute group, 7 (4.3%) in the subacute group, and 1 (3.0%) in the chronic group. The difference among the groups was not significant. However, the perioperative endoleak rate was significantly higher in the chronic group (18.1%) than in the acute (3.9%) and subacute (3.7%) groups (*P* = 0.02). Furthermore, the perioperative reintervention rate was significantly higher in the chronic group (12.1%) than in the acute (1.6%) and subacute (5.0%) groups (*P* = 0.03). The perioperative outcomes are summarized in Table 2.

**TABLE 2:** Procedure details and perioperative outcomes.

	Acute (*n* = 129) *N* (%)	Subacute (*n* = 161) *N* (%)	Chronic (*n* = 33) *N* (%)	*P*-value
Technical success	124 (96.1)	155 (96.3)	29 (87.9)	0.29
Endoleak	5 (3.9)	6 (3.7)	6 (18.1)*	**0.02**
Length of stay, day	15.1 ± 1.4	15.0 ± 1.7	15.5 ± 2.1	0.32
**Adjunctive procedure**				
Hybrid technique	1 (0.8)	2 (1.3)	1 (3.1)	0.43
Chimney technique	10 (7.8)	10 (6.2)	2 (6.2)	0.45
Single-branch S/G	4 (3.1)	4 (2.5)	1 (3.1)	0.56
**Perioperative outcomes**				
30-day mortality	3 (2.3)	0	0	0.34
Reintervention	2 (1.6)	8 (5.0)	4 (12.1)*	**0.03**
Major morbidity	22 (17.3)	26 (16.1)	6 (18.2)	0.43
Visceral malperfusion	3 (2.3)	4 (2.5)	0	0.56
Cerebral infarction	6 (4.7)	7 (4.3)	1 (3.3)	0.43
MODS	10 (7.6)	12 (7.5)	4 (12.1)	0.29
Lower limb malperfusion	3 (2.3)	3 (1.9)	1 (3.0)	0.18

*MODS, multiple organ dysfunction syndrome.*

*Statistically significant difference between this group and either of the other groups. Boldface value, show P < 0.05.

### Late Outcomes

Computed tomography angiography imaging at 5-years follow-up data were available for 230 patients (96, 114, and 20 patients in the acute, subacute, and chronic groups, respectively). The mean follow-up was 78 ± 22 months (range, 36–101 months). All-cause mortality was not different among the three groups. In the acute group, one patient died of acute myocardial infarction 6 months after TEVAR, and one died of a ruptured dissecting aneurysm 41 months after TEVAR. Two patients in the subacute group died of causes unrelated to the aorta during follow-up. No patients in the chronic group died. Late endoleak occurred in 20 patients overall: 9 (9.4%) in the acute group, 9 (7.9%) in the subacute group, and 2 (10.0%) in the chronic group. Reintervention was required in 12 patients overall: 9 in the acute/subacute group and 3 in the chronic group. The reintervention rate was significantly higher in the chronic group [2/20 (10%), *P* = 0.02]. Lower limb malperfusion occurred in five patients: one (1.0%) in the acute group, two (1.8%) in the subacute group, and two (10.0%) in the chronic group. The difference among the groups was significant (*P* = 0.03). The late outcomes are summarized in Table 3.

**TABLE 3:** Follow up results and aortic remodeling after TEVAR.

	Acute (*n* = 96) *N* (%), or mean ± SD	Subacute (*n* = 114) *N* (%), or mean ± SD	Chronic (*n* = 20) *N* (%), or mean ± SD	*P*-value
Follow-up, MoReinterventionMortality	47 ± 21 7 (7.3) 2 (2.1)	42 ± 23 3 (2.6) 2 (1.8)	41 ± 21 2 (10)* 0	0.27 **0.02** 0.54
Endoleak	9 (9.4)	9 (7.9)	3 (10)	0.67
Cerebral infarction	6 (6.3)	6 (5.3)	1 (5)	0.78
Lower limb malperfusion	1 (1.0)	2 (1.8)	2 (10)*	**0.03**

*Statistically significant difference between this group and either of the other groups. Boldface value, show P < 0.05.

### Aortic Remodeling

**FIGURE 2:**
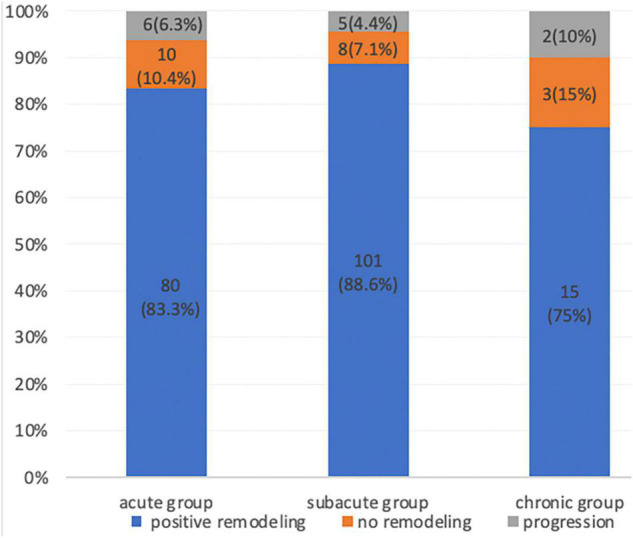
Proportion of patients with aortic remodeling (positive remodeling, no remodeling, and progression) during the CTA follow-up after thoracic endovascular aortic repair.

Aortic remodeling was analyzed using imaging data from the 230 patients with CTA at 5-years follow-up. The TL diameter increased, and the FL diameter decreased after TEVAR in all patients. An increase in the TL diameter was found in all three patient groups. The TL and FL diameter changes at the proximal thoracic aorta 2 cm below the LSA ostium, tracheal protuberance, and diaphragm were significantly greater in the acute and subacute groups than in the chronic group (*P* < 0.05). In addition, the TL diameter increase was significantly greater in the acute and subacute groups than in the chronic group. Complete obliteration of the FL with good remodeling in the stented thoracic aorta was achieved in 196 (85.2%) patients: 80 (83.3%) in the acute group, 101 (88.6%) in the subacute group, and 15 (75%) in the chronic group. FL thrombosis occurred in 90 (93.7%) of acute group, 109 (95.6%) of subacute group, 18 (90%) of chronic group. Although a trend toward a higher FL thrombosis rate was found in the acute and subacute groups, the difference was not significant (Figure 2; *P* = 0.2).

The relative diameter changes in the acute, subacute, and chronic groups are summarized in Table 4. The relative area changes on the acute, subacute, and chronic groups are showed in Table 5.

**TABLE 4:** Changes in the diameter of TL, FL, and aorta (mm) at 5-year follow-up.

	Acute	Subacute	Chronic	*P*-value
		**TL**		
Level A	16.3 ± 3.9	16.5 ± 5.4	13.0 ± 4.1*	**0.03**
Level B	13.1 ± 4.5	13.3 ± 3.4	10.1 ± 4.7*	**0.01**
Level C	12.1 ± 5.5	12.4 ± 4.6	9.1 ± 4.8*	**0.04**
Level D	4.4 ± 6.3	4.6 ± 5.5	4.2 ± 5.8	0.34
		**FL**		
Level A	–16.7 ± 4.7	–16.5 ± 5.8	–13.2 ± 4.1*	**0.01**
Level B	–12.8 ± 5.3	–13.0 ± 6.1	–10.4 ± 4.8*	**0.04**
Level C	–11.9 ± 5.1	–12.2 ± 4.9	–9.2 ± 5.3*	**0.03**
Level D	–5.5 ± 5.1	–5.5 ± 5.7	–4.5 ± 5.2	0.23
		**Aorta**		
Level A	–0.3 ± 0.9	–0.1 ± 1.4	–0.2 ± 1.1*	**0.01**
Level B	–0.3 ± 1.2	–0.4 ± 0.9	–0.5 ± 1.0*	**0.02**
Level C	–0.3 ± 0.5	–0.2 ± 0.8	–0.1 ± 0.9*	**0.04**
Level D	0.1 ± 0.7	0.1 ± 0.5	0.3 ± 1.1	0.53

*Statistically significant difference between this group and either of the other groups. Boldface value, show P < 0.05.

**TABLE 5:** Change in area of TL, FL, and aorta (mm^2^) at 5-year follow-up.

	Acute	Subacute	Chronic	*P*-value
		**TL**		
Level A	408.2 ± 130.1	328.3 ± 123.1	309.2 ± 120.1*	**0.02**
Level B	327.9 ± 132.3	307.1 ± 111.4	301.9 ± 162.3*	**0.03**
Level C	268.3 ± 154.9	238.5 ± 131.7	248. ± 152.9*	**0.04**
Level D	145.3 ± 139.8	155.1 ± 101.3	132.3 ± 119.8	0.49
		**FL**		
Level A	–412.8 ± 145.1	–422.3 ± 125.3	–402.8 ± 115.4*	**0.02**
Level B	–406.8 ± 167.8	–416.4 ± 147.5	–446.8 ± 152.6*	**0.03**
Level C	–334.2 ± 129.6	–334.6 ± 119.7	–314.6 ± 125.1*	**0.02**
Level D	–91.9 ± 110.7	–90.5 ± 105.2	–93.5 ± 119.2	0.83
		**Aorta**		
Level A	–33.4 ± 233.3	–34.5 ± 241.5	–34.5 ± 231.5*	**0.03**
Level B	–66.5 ± 156.5	–66.6 ± 1432.6	–63.6 ± 152.6*	**0.02**
Level C	–32.7 ± 172.7	–33.7 ± 145.7	–32.3 ± 123.6*	**0.03**
Level D	–21.9 ± 119.2	–26.2 ± 132.3	–26.5 ± 163.7	0.53

*Statistically significant difference between this group and either of the other groups. Boldface value, show P < 0.05.

## Discussion

The goals of TEVAR are complete coverage of the primary entry tear, prevention of aortic rupture, and promotion of reverse aortic remodeling. Although numerous studies have focused on post-TEVAR remodeling and clinical outcomes in patients with acute and chronic TBAD (3–7), few have examined patients with subacute dissection. One meta-analysis showed that the degree of remodeling was more consistent in acute than chronic dissection (10). Other studies showed that the capacity for reverse aortic remodeling after TEVAR was more pronounced for acute dissection (3–7). TEVAR promotes rapid expansion of the TL and collapse of the FL in the first 18 months after treatment (3). However, the dissected internal membrane is thinner and more fragile in the acute phase than in the subacute and chronic phases, which increases the risks of rupture, complications, and death. After 3 months, the dissection flap becomes thicker, straighter, and less mobile (11). Thus, the balance between fragility and plasticity might be clinically significant for the timing of TEVAR.

Optimal timing of treatment for TBAD remains controversial. The prospective VIRTUE Registry showed that aortic plasticity was greater in acute and subacute TBAD than in chronic TBAD (6). However, the study did not analyze clinical postprocedural differences because of the relatively small number of patients. Li et al. (12) reported that chronic dissection was associated with a lower rate of aortic remodeling than acute and subacute dissection; however, subacute dissection was associated with lower rates of major complications and mortality. The authors concluded that the optimal timing for TEVAR might be the subacute phase. Kato et al. (13) found significantly higher rates of early and late complications and mortality in patients with acute TBAD and suggested that TEVAR should be delayed beyond the acute stage. In another study by Li et al. (14), the long-term clinical outcomes and aortic remodeling after TEVAR were compared among patients with acute, subacute, and chronic TBAD. The authors found that patients with acute and subacute TBAD had an increased risk of rupture and complications at presentation, whereas those with chronic TBAD had an increased risk of aneurysmal dilation. Lee et al. (15) examined patients with acute, subacute, early chronic (<1 year), and late chronic TBAD and found that the maximal total aortic diameter and FL diameter were smaller in the acute/subacute group than in the early and late chronic groups. Survival free from major adverse aortic events after TEVAR was lowest in the late chronic group but was not significantly different between the acute/subacute and early chronic groups. The authors suggested that TEVAR should be performed within 1 year of symptom onset to achieve optimal aortic remodeling and safety. In our study, the outcomes after TEVAR were better in the acute and subacute groups than in the chronic group. The acute and subacute groups had lower rates of lower extremity malperfusion, cerebral infarction, and endoleaks and exhibited better aortic remodeling effects on CTA.

**FIGURE 3:**
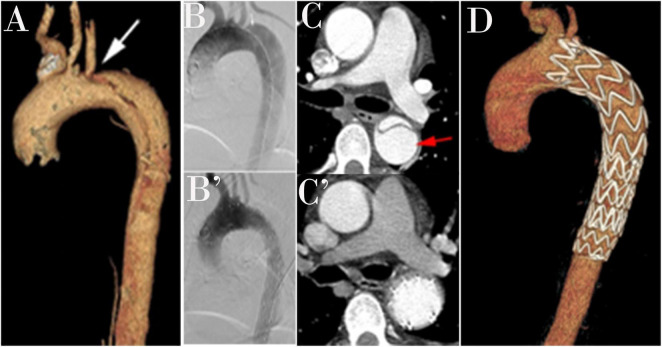
A three-dimensional computed tomography angiography (CTA) is shown in a patient with type B aortic dissection (TBAD; **A**). An intraoperative angiogram demonstrates TBAD **(B)**. A Post-operative angiogram after thoracic endovascular aortic repair (TEVAR; **B’**). Preoperative view with a patent false lumen **(C)**. False lumen obliteration after TEVAR **(C’)**. Completion angiogram after successful repair of TBAD shows the coverage of primary entry and no evidence of endoleak **(D)**.

Reduction in the FL and overall aortic diameters after TEVAR are major markers of aortic remodeling after dissection (Figure 3). In a systematic review, remodeling was more favorable in patients with acute dissection (80–90%) than in those with chronic dissection (38–91%; 10). The VIRTUE Registry showed that the FL thrombosis rate in the proximal and distal descending thoracic aorta was not significantly different among patients with acute, subacute, and chronic dissection (6). In our study, aortic remodeling was associated with a greater reduction in the diameter of the proximal descending aorta (proximal thoracic aorta 2 cm below the LSA ostium, tracheal protuberance, and diaphragm) than distal descending aorta (highest renal artery level). Although TEVAR stopped aortic expansion and enabled aortic reverse remodeling in stent coverage, favorable effects distal to the graft were not observed. We also found that the aortic diameter at the level of the renal artery increased after TEVAR. It is likely that treating the segment of the aorta above the diaphragm often did not address the main reentry tears. This might be explained by our treatment approach, which covers the proximal entry tear without covering all distal entry tears. This approach reduces the risk of SCI. The increase in the distal diameter was not significantly different among the three groups.

In the current study, we found that late morbidity and endoleak were more prevalent in the patients with chronic TBAD; however, all-cause mortality was similar, which differs from previous studies. Li et al. (15) found significantly lower 30-day and cumulative all-cause mortality rates as well as a lower major complication rate in patients with subacute TBAD. Nevertheless, data from the VIRTUE Registry showed similar all-cause and dissection-related mortality rates among patients with acute, subacute, and chronic TBAD, which agrees with our findings (6). Twenty (8.7%) of the 230 patients with CTA follow-up data developed a late endoleak; 12 endoleaks were type Ia and accepted additional stent graft implantation and balloon expansion. The apposition of self-expanding covered endografts to the aortic wall was temporarily incomplete for a short time after the procedure. Therefore, contrast material could flow into the FL through the microinterval between the endografts and the aorta. The other factor that contributed to Ia endoleak formation was incomplete stent graft apposition to the lesser curvature of the aortic arch, resulting in a bird beak defect. After the primary entry tear was sealed because of hemodynamic change, the space filled with thrombotic material. Type II endoleaks usually originated from either the intercostal arteries or the LSA. In our study, they most likely originated from the intercostal arteries. There was always blood filling the abdominal FL. When most thoracic branches were fed by FL blood flow, retrograde flow and incomplete thrombosis might have persisted because of the presence of adequate outflow tracts. Analysis of our morphological data revealed that the distinction among the three groups was statistically significant. These morphological changes indicated that aortic remodeling was less favorable in the chronic group than in the acute and subacute groups, but with no significant difference between the acute and subacute groups. Therefore, we might assume that persistent flow through an unsealed entry tear can delay thrombosis in the FL.

One of the most devastating complications following TEVAR is stent induced new entry (SINE; 16). Most stent graft were initially designed to treat thoracic aneurysmal disease, not dissection. In TEVAR, sealing of the stent graft required a length of normal proximal and distal aorta. Due to complexity of aorta dissection, both rigidity of stent graft and the degree of oversizing have been tended to contribute to SINE (17). Excessive radial force, especially when stent grafts were oversized more than 20% in relation to native aorta, have been reported associated with proximal SINE (18). Stent graft sizing is based on the size of the proximal landing zone, the sizing of distal diameter is often much smaller in the distal landing zone, resulting in much greater radial force of stent graft on the distal aortic wall, which has been proposed as one of the most important risks for distal SINE. In addition to the sizing and rigidity of TEVAR, the timing of TEVAR also contributed risk of SINE. Jang et al. reported that distal SINE was significantly more frequent in chronic dissection (19). As the incidence of this distal SINE, probably because the intimal membrane is thicker and more fibrotic and therefore less elastic and less able to remodel than in acute dissection (20). The use of tapered components and additional restrictive bare-metal stent in the treatment of TBAD may lead to decrease rates of distal SINE. Fortunately, there was no SINE in our study. The use of tapered (4 mm/8 mm) components may one of reasons that contribute to decrease rates of SINE in this study.

This study had several limitations. First, it was retrospective in design and was conducted in three centers. The sample size was relatively small, particularly for the chronic group, which precluded detailed analyses. Second, a number of patients had inconsistent follow-up scans, CTA data does not provide the visualization of dynamic remodeling process. Moreover, the mean follow-up was too short to fully evaluate aneurysmal degeneration in the non-stented part of the abdominal aorta. Therefore, it was not possible to fully examine the risk of reintervention. Third, our study included patients with uncomplicated TBAD, which might have introduced confounding bias. Future studies with a homogeneous study design, inclusion criteria, and outcome measures as well as a larger sample size and longer follow-up are warranted.

## Conclusion

Patients with acute, subacute, and chronic TBAD had different outcomes in this study. Patients with acute and subacute TBAD had fewer complications than those with chronic TBAD. Aortic remodeling after TEVAR was more favorable in patients with acute and subacute TBAD than in patients with chronic TBAD. TEVAR promoted more positive remodeling at the stent graft level than at the distal level of the aorta.

## Data Availability Statement

The original contributions presented in the study are included in the article/supplementary material, further inquiries can be directed to the corresponding authors.

## Ethics Statement

The studies involving human participants were reviewed and approved by the study was supported by Local Ethics Committee of Nanjing Drum Tower Hospital (Nanjing, China), The People’s Hospital of Changzhou (Changzhou, China), and Nanjing First Hospital (Nanjing, China). The patients/participants provided their written informed consent to participate in this study.

## Author Contributions

GY, HG, GW, YZ, LZ, MZha, XL, and MZho: conception and design, literature search, writing the manuscript, and critical revision of the manuscript. All authors read and approved the final version of the manuscript.

## Conflict of Interest

The authors declare that the research was conducted in the absence of any commercial or financial relationships that could be construed as a potential conflict of interest.

## Publisher’s Note

All claims expressed in this article are solely those of the authors and do not necessarily represent those of their affiliated organizations, or those of the publisher, the editors and the reviewers. Any product that may be evaluated in this article, or claim that may be made by its manufacturer, is not guaranteed or endorsed by the publisher.
